# Enhancing Quartz Infrared Absorption by Tuning Femtosecond Laser Surface Texturing Patterns

**DOI:** 10.3390/ma19132810

**Published:** 2026-07-02

**Authors:** Isabella Petruzzellis, Raffaele De Palo, Andrea Zifarelli, Pietro Patimisco, Felice Alberto Sfregola, Stefania Caragnano, Caterina Gaudiuso, Francesco Paolo Mezzapesa, Vincenzo Spagnolo, Antonio Ancona, Annalisa Volpe

**Affiliations:** 1 Chemistry Department, Università degli Studi di Bari, Via Orabona 4, 70126 Bari, Italy; i.petruzzellis1@studenti.uniba.it; 2 Physics Department, Politecnico di Bari, Via Amendola 173, 70125 Bari, Italy; vincenzoluigi.spagnolo@poliba.it (V.S.); annalisa.volpe@poliba.it (A.V.); 3 Physics Department, Università degli Studi di Bari, Via Amendola 173, 70125 Bari, Italy; andrea.zifarelli@uniba.it (A.Z.); pietro.patimisco@uniba.it (P.P.); felice.sfregola@uniba.it (F.A.S.); s.caragnano@phd.uniba.it (S.C.); antonio.ancona@uniba.it (A.A.); 4 PolySense Innovations srl, Via Amendola 173, 70125 Bari, Italy; 5 Institute for Photonics and Nanotechnologies (IFN), National Research Council, Via Amendola 173, 70125 Bari, Italy; caterina.gaudiuso@cnr.it (C.G.); francescopaolo.mezzapesa@cnr.it (F.P.M.)

**Keywords:** quartz, femtosecond laser, blackening

## Abstract

Quartz is widely employed in optoelectronic and sensing applications owing to its excellent mechanical and chemical properties. However, its intrinsic transparency up to 5 μm limits its direct use as a photodetection substrate across the near- and mid-infrared spectral regions. Laser surface texturing for the fabrication of the so-called black quartz represents a promising strategy to overcome this limitation. In this work, different femtosecond (fs) laser texturing strategies were investigated on a 1 mm thick α-quartz wafer, namely uniform milling, grid-patterned grooves, and localized arrays of ablated craters. The fs-laser-treated quartz samples showed a transmittance reduction of up to 60% within the quartz transparency window in the infrared range, with crater matrices providing the most effective blackening performance. The enhanced absorption was attributed to light-trapping effects induced by the tapered crater geometry, which promotes multiple internal reflections and increased optical confinement within the substrate. The proposed strategy demonstrates a reliable, maskless, and chemical-free surface functionalization strategy for the fabrication of quartz-based substrates for broadband infrared photodetection in sensing applications.

## 1. Introduction

Quartz, the crystalline form of silicon dioxide (SiO_2_), is the second most abundant mineral found on Earth and one of the most widely used materials in the fields of optoelectronics, photovoltaics, optical fiber communication, and sensing, owing to its outstanding optical, thermal, and chemical properties [1]. With wide indirect and direct bandgaps (6.3 eV and 9.2 eV, respectively), quartz shows high optical transmission across the UV–IR spectral range, along with a low thermal expansion coefficient, high piezoelectricity, chemical stability, electrical insulation, wear resistance, and oxidation resistance.

In sensing applications, quartz has been widely employed for electrochemical sensors based on Quartz Crystal Microbalance (QCM) [2], as well as in Quartz-Enhanced Photoacoustic Spectroscopy (QEPAS) [3] and Light-Induced Thermoelastic Spectroscopy (LITES) [4], typically used in the form of Quartz Tuning Forks (QTFs). In LITES, QTFs operate as infrared photodetectors by converting the incident radiation into an electrical signal via the piezoelectric effect of quartz, following light transmission through a gas absorption cell. Since QTF-based photodetection relies on radiation absorption, its spectral response is strongly limited at wavelengths below approximately 5 μm because of the high transparency of quartz in the near- and mid-infrared spectral range. Thus, enhancing the optical absorption of quartz in this range is essential for extending its applicability for near-infrared photodetection.

Surface functionalization of quartz by means of nano- and micro-structures introduction on the material surface represents an attractive solution for more efficient QTF-based photoconductive applications in different spectral regions. The blackening approach consists of enhancing the optical absorptance of a material within spectral regions where it is naturally transparent, without significantly affecting its intrinsic electronic properties [5,6]. This approach, also referred to as blackening, has been widely explored in photovoltaics [7,8,9], whereas its application to infrared photodetection remains relatively unexplored. For application in optical communication, imaging, and spectroscopy, black materials must also exhibit additional features such as surface durability, material robustness, reusability, and scalable manufacturability. Nonetheless, hardness and brittleness are also intrinsic properties of quartz, which make it a challenging material for micromachining due to how it easily undergoes cracking and chipping during material processing.

Quartz surface texturing can be realized through various techniques, including diamond cutting [10], photolithography, wet and dry etching [11,12,13], and ion beam etching [14]. Despite their widespread use, these techniques suffer from several drawbacks, such as chemical contamination, limited processing resolution, and long processing times. In this context, femtosecond laser irradiation represents a convenient and reliable alternative [15] for inducing controlled modifications both on the surface and within the bulk of quartz [15,16,17,18,19,20,21].

Laser surface texturing is a direct, one-step fabrication method used to create features on micro- and nano-scales for functionalizing a broad range of material surfaces, enabling the tailoring of optical, mechanical, or chemical properties [19,22,23,24]. Depending on the desired texture, geometry and characteristic feature dimension, several laser-based techniques can be exploited, ranging from the micrometer-scale Direct Laser Writing (DLW) [25] to sub-micrometer Laser-induced Periodic Surface Structures (LIPSS) [26] and down to a few hundred nanometers achieved by Direct Laser Interference Patterning (DLIP) [27]. Compared with other surface treatment techniques [27], which often require toxic chemical reagents, expensive mask fabrication, or complex production systems, femtosecond laser processing offers a cleaner and more flexible alternative. These advantages, combined with the capability of providing high-resolution surface modification, have driven extensive investigations on femtosecond (fs) laser processing as an alternative method for material surface engineering [28,29]. Significant progress has been achieved in fs-laser surface texturing for tailoring the mechanical, electrical, and optical properties of various materials, including quartz, by exploiting the extreme flexibility and sub-micrometric precision of fs-laser pulses. Despite these advances, the development of application-specific blackened materials remains challenging, and most recent studies on laser blackening have mainly focused on silicon [8,30,31,32,33,34,35] and diamond [36,37,38], with most investigations still considering processing in heavily doped and low-pressure atmospheres, resulting in increased process complexity and environmental footprint. Recently, De Palo et al. [39] demonstrated the effectiveness of fs-laser blackening of quartz as a strategy to enhance its optical absorption without requirements for a controlled and doped atmosphere, resulting in lower environmental impact and an easier overall process. However, although black quartz has been obtained through arrays of irradiated spots or localized LIPSS patterns, a systematic comparison with other laser-textured morphologies commonly employed in materials processing is still lacking [22,40,41,42].

Thus, in this work, alternative laser-texturing strategies for quartz blackening were investigated. Different texturing patterns were explored, including laser milling over localized areas, grid patterns of ablated grooves, and localized arrays of ablated craters with different depths. Texturing was performed in an ambient atmosphere, thus providing a single-step and environmentally friendly blackening strategy. The optical response of the fabricated blackening patterns was characterized by measuring their transmittance spectra across the near- to mid-infrared (NIR–MIR) spectral range.

## 2. Materials and Methods

The quartz wafers employed in this study, supplied by Nano Quartz Wafer GmbH (Langenzenn, Germany), consist of double-polished z-cut quartz wafers with dimensions of 24.5 × 24.5 × 1 mm.

### 2.1. Experimental Apparatus for Femto-Laser Texturing

**Figure 1:**
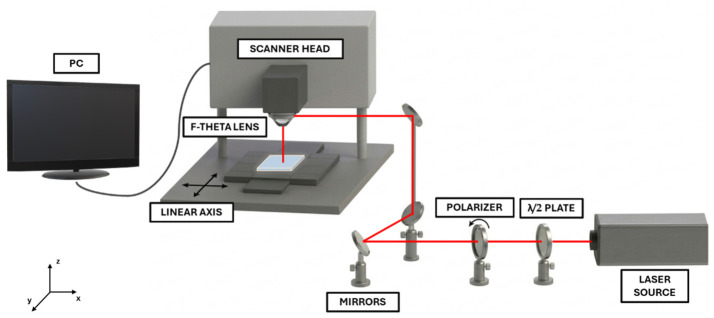
Schematic representation of the apparatus for laser texturing of quartz wafers.

Figure 1 shows the experimental setup based on a laser source with a solid-state active medium in Yb:YAG, the Pharos SP 1.5 from Light Conversion (Vilnius, Lithuania). The source operates with a chirped pulse amplification (CPA) system, a Mode-Locking oscillator and a Pulse Picker capable of expanding and compressing the pulse duration and emitting ultrashort pulses with a tunable temporal duration *τ_P_*. During the investigation, the pulse duration was fixed at 200 fs. The emitted beam had an emission wavelength of 1030 nm with a Gaussian-like profile with a quality factor *M*^2^ = 1.3. A maximum power of 6 W, a maximum pulse energy of 1.5 mJ, and a repetition frequency *f_R_* were tunable from a single pulse up to 1 MHz. The laser beam was emitted from the source with linear polarization and was first directed onto a *λ*/2 half-wave plate and a polarizer, which transmitted the P-component of polarization, allowing the adjustment of the beam power. Finally, through a system of mirrors, the laser beam was directed to a digital head scanner AGV-HP Nmark from Aerotech (Pittsburgh, PA, USA), employed with a 160 mm focal length F-theta lens. The estimated laser diameter in air was 32 μm. The samples were placed in the focal plane of the F-theta lens on air-bearing linear stage ABL1500, Aerotech, allowing fine linear translational movements of the samples. Both the head scanner and the stage were controlled by the A3200 Motion Composer software (5.02.000 version), ensuring synchronized movements and correct selection of the working area.

All texturing processes were performed in air under ambient conditions. After fs-laser processing, the samples were cleaned with isopropyl alcohol in an ultrasound bath for 3 min to remove the processing residues. The morphological analysis of the superficial patterns was performed using a Nikon Eclipse ME600 optical microscope (Melville, NY, USA) equipped with a digital camera interfaced with NIS Elements software (5.21.00 version) and a Keyence digital optical microscope (Osaka, Japan).

### 2.2. Fabrication of Milling and Grid Texturing Patterns

**Figure 2:**
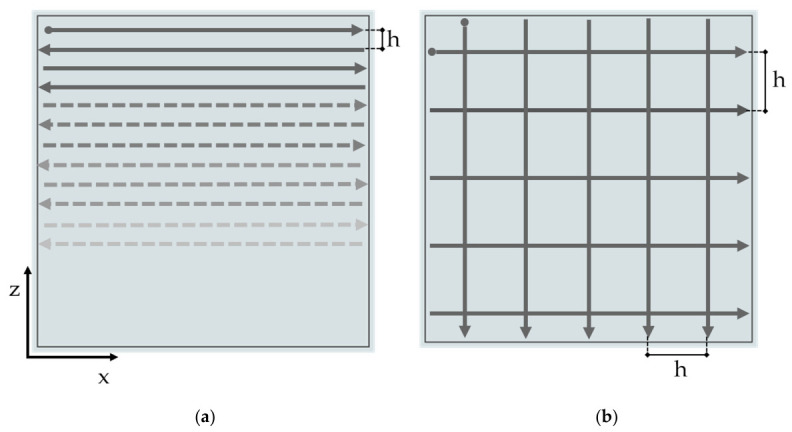
A schematic of the laser scanning strategy employed during (**a**) milling and (**b**) grid texturing of quartz surface wafers; the hatch *h* is the distance between two consecutive scanning lines.

The first quartz-blackening strategies investigated were the milling and grid patterns. The milling was performed by superimposing a single parallel scanning pattern with a hatch distance of *h* = 16 μm, as shown in Figure 2a. Considering the laser beam diameter of 32 μm, *h* = 16 μm resulted in 50% overlap between adjacent scanning, thus achieving a uniform ablated milling area; the grid, instead, was realized by superimposing two perpendicular single scanning patterns (one horizontal and one vertical), with a hatch distance *h* of 125 μm, as shown in Figure 2b. This strategy was chosen to create structures with a uniform surface density *D_S_* of 40% across the ablated area. Each experiment was performed over a target area of dimensions 11 × 11 mm^2^.

The laser repetition rate and the pulse energy were fixed at *f_R_* = 5 kHz and *E_P_* = 90 μJ, respectively, on both patterns, while the scan speed was set to $v_{s,milling}=15\;mm/s$ for the milling pattern and $v_{s,grid}=13\;mm/s$ for both the scanning directions of the grid pattern. According to previous studies by De Palo et al. [17,40] on the ablation threshold fluence of quartz *Φ_th_*, these values were chosen to be well above the ablation threshold in order to obtain the desired surface texturing. The working parameters for each pattern are summarized in Table 1.

### 2.3. Fabrication of Matrices of Craters Patterns

Localized arrays of ablated craters were also investigated. Two crater series were fabricated using different numbers of laser pulses, *N* = 50 and *N* = 100. A preliminary study was carried out to determine the optimal center-to-center spacing, *h*, between adjacent craters, with the aim of maximizing the surface coverage while preserving the mechanical integrity of the quartz substrate. The maximum density was set to 40%, as higher values resulted in increased surface damage due to the propagation of structural defects and cracks. The achieved coverage density was subsequently verified through post-processing digital image analysis of the optical micrographs using the ImageJ software (1.54q version).

The working parameters used to achieve the target coverage density for both the *N* = 50 and *N* = 100 crater matrices are listed in Table 2.

**Figure 3:**
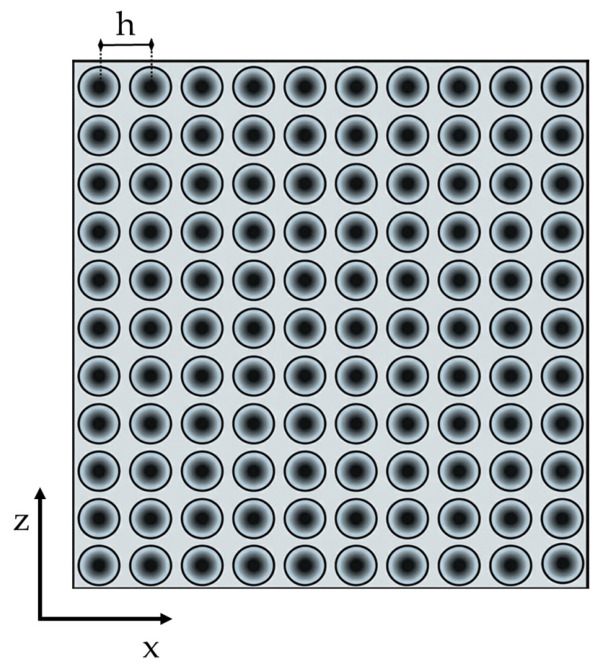
A schematic of the laser scanning pattern employed for laser texturing of craters’ matrices.

Accordingly, both crater matrices were designed using a square array pattern over an area of 11 × 11 mm^2^. For both structures, the laser repetition rate and the pulse energy were fixed at *f_R_* = 1 kHz and *E_P_* = 37.5 μJ, respectively. These values were selected to remain well above the ablation threshold of quartz and to obtain the desired surface texturing, in agreement with previous studies [17,19,40]. A schematic of the laser scanning strategy employed for l for the fabrication of crater matrices on quartz wafers is illustrated in Figure 3.

## 3. Results and Discussion

### 3.1. Morphological Characterization of Milling and Grid Texturing Patterns

**Figure 4:**
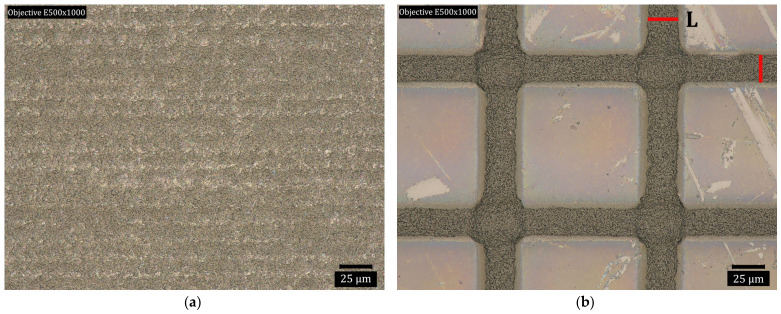
Micrographs of (**a**) the milling pattern and (**b**) the grid pattern obtained through laser irradiation *f_R_* = 1 kHz and *E_P_* = 90 μJ.

The textured quartz wafers were optically characterized to assess the morphological properties of the fabricated structures, such as the ablation depth, the surface roughness *R_s_* for the milling and grid pattern, and the characteristic structure width *L*. Representative optical micrographs of the two patterns are shown in Figure 4.

**Figure 5:**
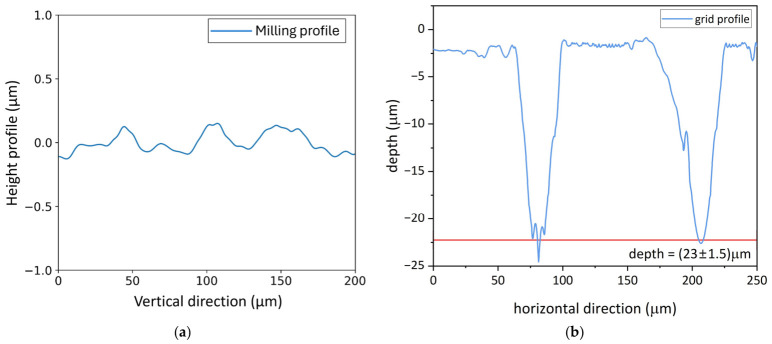
Reconstruction of (**a**) milling line profile in direction perpendicular to the laser scanning direction and (**b**) grid profiles in the horizontal direction.

The ablation depth *d* and the taper angle *ϑ*, defined as the inclination of the ablated structures’ sidewalls with respect to the surface normal, were estimated by reconstructing the cross-sectional profiles of the textured features using the digital microscope. Representative topographical profiles for both the grid-patterned features measured along the horizontal direction and the line profile of the milling pattern perpendicularly to the scanning direction are shown in Figure 5.

From the optical characterization, the morphological parameters of the textured features were determined together with their associated uncertainties, as reported in Table 3. In the case of milling texturing, the surface roughness *R_s_* is reported. The uncertainties were calculated as the standard deviation of the mean values.

### 3.2. Morphological Characterization of Matrices of Craters Patterns

**Figure 6:**
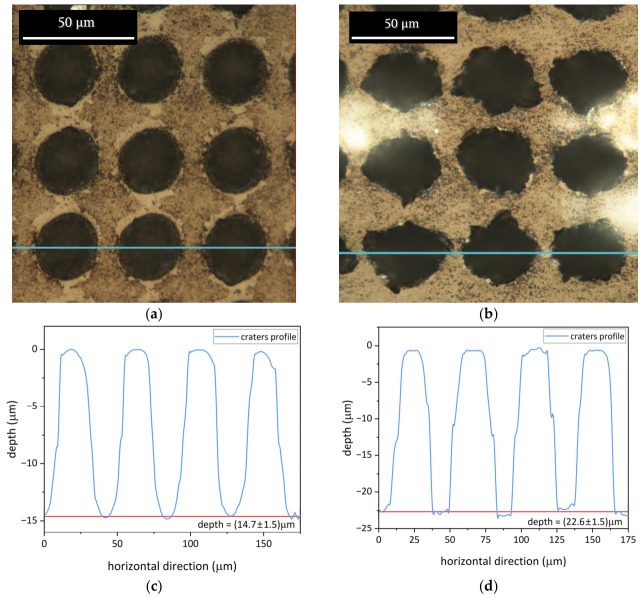
Micrographs acquired with the digital microscope of the two matrices for (**a**) *N* = 50 and (**b**) *N* = 100 obtained through laser irradiation at *f_R_* = 1 kHz and *E_P_* = 37.5 μJ; reconstruction of craters profile for (**c**) *N* = 50 and (**d**) *N* = 100. The blue line represents the direction in which the profiles have been reconstructed. The red line represents the depth of the craters.

The craters’ morphology was characterized by using the digital microscope to measure the geometrical characteristics of the ablated craters, namely the ablation depth d, the diameters *D*, and the taper angle *θ*. Representative optical micrographs are shown in Figure 6a,b. Moreover, the cross-sectional profiles were reconstructed from the microscope measurements. The resulting topographical profiles for crater structures are reported in Figure 6a,b, where the blue lines indicate the directions along which the profiles were extracted.

As shown in Figure 6, fs-laser texturing induces significant morphological modifications together with the formation of surface and sub-surface defects in quartz wafers with amorphization and the formation of polycrystalline structures [41,42]. In particular, repeated laser irradiation led to a greater formation of polycrystalline defects a few micrometers beneath the surface in the crater matrices realized with a higher number of pulses *N* [17].

The morphological parameters obtained from the optical characterization, along with their associated uncertainties, are reported in Table 4. The uncertainties were calculated as the standard deviation of the mean values. The ablation depth was estimated from the reconstructed cross-sectional profiles’ crater profiles.

A correlation can be observed between the number of laser pulses *N* and the taper angle. For *N* = 50, the craters exhibit a more conical profile, while for *N* = 100, a smaller taper angle and steeper crater sidewalls are observed as a result of cumulative pulse irradiation.

### 3.3. Optical Characterization of Blackened Quartz Wafers

**Figure 7:**
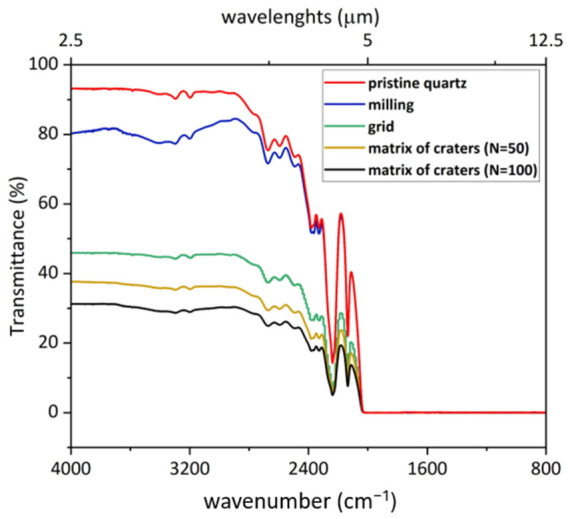
Transmittance spectra of all textured quartz samples measured using a Fourier transform spectrometer: (i) red line, pristine quartz slab; (ii) blue line, milling pattern; (iii) green line, grid pattern; (iv) yellow line, craters matrix realized with *N* = 50 pulses; and (v) black line, craters matrix realized with *N* = 50 pulses.

The effectiveness of fs-laser *blackening* in reducing the quartz transmittance within its intrinsic transparency window in the infrared region was evaluated through optical transmission measurements using a Nicolet iS50 FTIR spectrometer (ThermoFisher Scientific, Waltham, MA, USA). Spectral acquisitions were performed over the mid-infrared range from 4000 to 800 cm^−1^, corresponding to wavelengths between 2.5 and 12.5 μm, for both pristine quartz and the laser-textured substrates fabricated with milling, grid and craters matrices patterns. This spectral region was specifically selected to emphasize the transition in optical behavior of pristine quartz occurring around 5 μm, where the material changes from highly transparent to strongly absorbing. The resulting transmittance spectra are reported in Figure 7, providing a direct comparison of the optical responses associated with the different surface morphologies induced by laser texturing.

The spectral measurements revealed that the textured area did not alter the transmittivity of the quartz crystal for wavelengths longer than ~5 μm (wavenumbers < 2000 cm^−1^). Conversely, all laser-textured samples exhibited a significant reduction in transmittance for wavelengths shorter than ~5 μm (wavenumbers > 2000 cm^−1^) compared with pristine quartz. Among the investigated surface morphologies, the crater matrix approach proved to be the most effective texturing strategy for reducing transmittance and thus enhancing optical absorption. While the milling pattern produced only a moderate reduction in transmittance, the crater matrices achieved a much stronger blackening effect. The observed behavior can be attributed to an enhanced light-trapping effect [34,43], governed by the relationship between the characteristic dimensions of the textured surface and the incident wavelength. In the crater matrices, the crater diameter and center-to-center spacing are significantly larger than the target wavelengths so that the interaction can be described within the framework of geometrical optics. The inclined crater sidewalls redirect part of the incident radiation inside the substrate at angles compatible with total internal reflection, increasing the effective optical path length and, consequently, the absorption probability. Crater density therefore plays a dominant role, as it determines the fraction of radiation intercepted and redirected by the textured surface. Indeed, despite the comparable depth achieved by the grid pattern and the *N* = 100 crater matrix, the former demonstrated itself to be less effective in enhancing the infrared absorption. By contrast, the milling pattern is geometrically characterized by the surface roughness *R_s_*, which has a dimension smaller than the IR wavelengths, resulting in an ineffective light-trapping effect of the incident radiation. This observation suggests that the light-trapping effect is governed more strongly by the overall surface morphological properties than by the depth alone of the characteristic texture feature. In particular, the tapered crater geometry promotes multiple internal reflections and more efficient confinement of the incident radiation within the quartz substrate [43,44]. A clear correlation was observed between the number of pulses *N* and the optical response. As summarized in Table 4, both crater depth and diameter increased with *N*, reaching a maximum depth of 22.1 μm for the *N* = 100 craters’ matrix, due to the accumulation of laser-induced structural defects. Despite the appearance of surface cracks and chipping phenomena, the high-density crater matrices, particularly for the *N* = 100 matrix, exhibited the lowest transmittance, approximately 30% lower than that of pristine quartz across the investigated spectral range. In this framework, scattering and reflection losses were taken into account when interpreting the measured transmittance reduction. These contributions are considered negligible with respect to the light-trapping mechanism discussed above, within the scope of the present comparative study. Indeed, light-trapping mechanisms have been widely reported to enhance optical confinement and increase the effective optical path length inside textured materials, thus increasing the probability of absorption while limiting the relative impact of scattering losses [43,44,45]. Regarding reflection losses, useful insights can be drawn from the optical properties of quartz. In the 1–5 μm spectral range, the reflectance of pure quartz is generally below 10% [46], whereas the transmittance variations observed after laser texturing are considerably larger, as shown in Figure 7. Therefore, reflection losses alone cannot account for the measured reduction in transmittance. Moreover, surface blackening is generally associated with a reduction in reflectance, which further supports the interpretation that reflection is not the dominant contribution to the observed optical response. Thus, even if the present transmittance measurements do not allow for an absolute determination of all the optical components of the laser-textured surfaces, the large transmittance reduction observed is consistent with enhanced light trapping and increased absorption probability.

These results indicate that crater-based texturing, investigated in this paper, turned out to be an effective blackening strategy for extending the infrared absorption capability of quartz.

## 4. Conclusions

In this work, the fabrication of black quartz through ultra-fast laser surface texturing of a 1 mm thick quartz wafer was systematically investigated. The main objective was to overcome the intrinsic transparency of quartz in the near- and mid-infrared spectral ranges and thereby enhance its infrared absorption through fs-laser surface texturing [17,19,41]. Three different texturing strategies were explored and comparatively characterized, namely area milling, grid-patterned ablated grooves, and localized crater matrices. Among the investigated morphologies, the crater matrix approach proved to be the most effective strategy. By optimizing both the number of laser pulses per spot (*N* = 50 and *N* = 100) and the hatching distance to achieve a surface coverage density of 40%, a significant reduction in quartz optical transmittance of up to approximately 60% with respect to pristine quartz was achieved. The enhanced absorption by the black quartz is attributed to light-trapping effects induced by the textured crater morphology, which promotes multiple internal reflections and increases the optical path length of the radiation confined within the quartz substrate.

Overall, the results demonstrate that fs-laser texturing is a reliable and versatile approach for the fabrication of black quartz with enhanced infrared absorption. The proposed structures represent promising candidates for enhanced infrared photodetection for sensing applications requiring efficient optical absorption over a broad infrared spectral range.

## Figures and Tables

**Table 1:** Working laser parameters for milling and grid texturing strategies. Fluence and laser repetition rate were fixed: *f_R_* = 5 kHz and *E_P_* = 90 μJ for both the patterns.

	v*_scan_* (mm/s)	*h* (μm)	D_S_ (%)
milling	15	14	100%
grid	13	125	40%

**Table 2:** Working laser parameters for matrix texturing strategies. Pulse energies and laser repetition rate were fixed at *E_P_* = 37.5 μJ and *f_R_* = 1 kHz for both *N* values.

	*h* (μm)	D_S_ (%)
*N* = 50	44	40
*N* = 100	43	40

**Table 3:** Morphological characteristics of the milling and grid patterns.

	*d* (μm)	*ϑ* (°)	R_s_ (μm)
milling	3.0 ± 0.5	\	0.3 ± 0.1
grid	23 ± 1.5	8 ± 3	\

**Table 4:** Geometrical characteristics of both the textured matrices with craters at *N* = 50 and *N* = 100.

	*D* (μm)	*d* (μm)	*ϑ* (°)
*N* = 50	34.2 ± 3.1	14.7 ± 1.5	50 ± 6
*N* = 100	35.3 ± 4.0	22.6 ± 1.5	25 ± 5

## Data Availability

The original contributions presented in this study are included in the article. Further inquiries can be directed to the corresponding author.
